# COVID-19-Induced Phlegmasia Cerulea Dolens

**DOI:** 10.7759/cureus.33644

**Published:** 2023-01-11

**Authors:** Stephanie Cohen, Joshua Lynch

**Affiliations:** 1 Emergency Medicine, University at Buffalo Jacobs School of Medicine and Biomedical Sciences, Buffalo, USA

**Keywords:** deep vein thrombosis (dvt), sars-cov2, covid-related hypercoagulability, covid-19, phlegmasia cerulea dolens

## Abstract

A 44-year-old male with a history of deep venous thrombosis (DVT) and pulmonary embolism (PE) with the inferior vena cava (IVC) filter in place and peripheral vascular disease (PVD) status post lower extremity vascular stenting presented from a COVID-19 rehabilitation center with bilateral phlegmasia cerulea dolens and no palpable popliteal or dorsalis pedis pulses, at risk for venous gangrene and loss of limbs. The patient was anticoagulated and taken emergently to the operating room for vascular surgery where thrombolysis with alteplase and mechanical thrombectomy were performed. Bilateral thrombolysis infusion catheters were placed for two days. The patient had a return of arterial signals in the feet and decreasing clot burden. The patient is expected to make a full recovery.

## Introduction

COVID-19 has been widely studied since its outbreak in Wuhan, China, in December 2019. As of December 16, 2022, there have been more than 647 million confirmed cases of COVID-19 and more than six million deaths [1].

Eighty-one percent of patients with COVID-19 have mild symptoms (no symptoms or mild pneumonia), 14% of patients have severe disease (dyspnea, hypoxia, or rapid onset of multifocal pneumonia), and 5% of patients have a critical disease (respiratory failure, shock, or multiorgan dysfunction) [2]. The most common presenting symptoms are cough and fever, followed by myalgia, headache, dyspnea, sore throat, diarrhea, nausea, vomiting, and loss of taste or smell.

Many patients with COVID-19 have an elevated D-dimer and are hypercoagulable, thought to result from endothelial injury [3]. Venous thromboembolism (VTE) is very common in acutely ill patients with COVID-19, seen in up to one-third of patients in the ICU, even when on prophylactic anticoagulation. A small study showed that the incidence of acute limb ischemia has increased since the start of the COVID-19 pandemic [4].

A case report recently documented a 12-year-old girl who had tested positive for SARS-CoV-2 and presented with phlegmasia cerulea dolens with venous gangrene complicated by massive pulmonary embolism (PE) requiring emergent mechanical thrombectomy and extracorporeal membrane oxygenation [5]. With the emerging cases and data on hypercoagulability in COVID-19 patients, it is important to be increasingly aware of potential complications when caring for a patient who has tested positive for COVID-19 or in patients who are under suspicion.

## Case presentation

**Figure 1:**
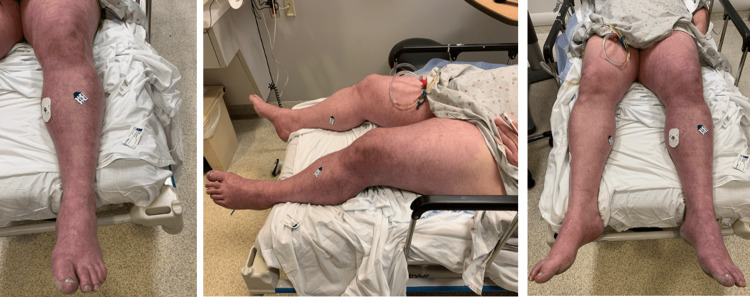
Clinical photograph of the patient's extremities.

A 44-year-old male with a past medical history significant for ulcerative colitis status post colectomy, PE/deep vein thrombosis (DVT) with inferior vena cava (IVC) filter placement a few years prior, peripheral arterial disease (PAD) treated with lower extremity vascular stenting, newly diagnosed diabetes mellitus, and COVID-19 (IgG antibody positive) presented from a COVID-19 rehabilitation center for evaluation of worsening shortness of breath, abdominal pain, back pain, and left lower extremity pain. The patient stated that he had worsening shortness of breath for the past few days but woke up the morning of the presentation with abdominal pain, sciatic back pain, and excruciating left lower extremity pain. He reported that he had no sensation in his left lower extremity distal to the knee. At presentation, the patient had a heart rate of 145 beats per minute, respiratory rate of 26 breaths per minute, saturation of 99% on 15 L/minute of oxygen via a non-rebreather mask, blood pressure of 88/42 mmHg, and an oral temperature of 36.7 °C. On examination, the patient had edema and mottling of the bilateral lower extremities, left greater than right (Figure 1).

He was found to have no Dopplerable pulse at bilateral dorsalis pedis arteries but did have biphasic pulses at bilateral femoral arteries. The patient underwent CT angiography of the torso, which showed normal arterial flow to the limbs, but distended IVC below the filter, consistent with IVC occlusion. The patient was started on a continuous heparin infusion. Duplex sonography confirmed extensive DVTs bilaterally. After consultation with the vascular surgery service, the patient was diagnosed with bilateral phlegmasia cerulea dolens with a risk for venous gangrene and loss of limb.

The patient was taken to the operating room emergently, where intraoperative central and bilateral lower extremity venograms were obtained. The AngioJet™ (Boston Scientific, Marlborough, MA, USA) device and sheath were passed from the left leg into the central venous circulation. The device was engaged and performed thrombolysis on the left with its functional element residing in the distal IVC adjacent to the filter (5 mg alteplase was infused). The device was withdrawn, and the wire was then passed up the right leg into the distal central circulation and 5 mg alteplase was infused. The device was withdrawn with its wires left in place. The alteplase was left to dwell in the distal IVC for 12 minutes. The device was then introduced first on the left and then on the right in thrombectomy mode and dissolved clots were suctioned. Cragg-McNamara™ (Medtronic, Minneapolis, MN, USA) catheters were then placed bilaterally, and the infusing tips were left adjacent to the IVC filter in the distal vena cava. The patient was transferred to the medical intensive care unit with ongoing bilateral thrombolysis. After the procedure, the patient had patent common iliac and external iliac veins bilaterally, with a moderate burden of thrombus surrounding the IVC filter in the mid-to-distal IVC. After the procedure, the patient had a return of arterial signals to the foot. On postoperation day (POD)-1, the patient had repeat venograms, which showed improvement of thrombus burden, but residual thrombus was present at the distal IVC and iliac bifurcation. The patient underwent catheter removal on POD-2. The patient has remained hemodynamically stable through his hospital course, has regained sensation in the lower extremity, and is expected to make a full recovery. 

## Discussion

Phlegmasia is derived from Greek for *inflammation*; cerulea is from cerulean, which in Latin means *dark blue*; and dolens comes from Latin and denotes *suffering*. Therefore, in combination, phlegmasia cerulea dolens describes a painful discoloration that comes from inflammation. The tissue ischemia results from inflammation, causing increased interstitial pressure in the limb [6]. This can result in compartment syndrome [7] or, worse, limb gangrene [8].

The aforementioned case demonstrates one of the many complications of COVID-19. Numerous studies have shown an increase in thromboembolic events in patients who have tested positive for COVID-19 [3-5,9]. Additional research still needs to be conducted concerning hypercoagulability in this patient population. It would be beneficial to understand if prophylactic anticoagulation in these patients would decrease the incidence of thromboembolic events. A recent study showed that there is still a high incidence of VTE/PE in patients who are both prophylactically and therapeutically treated with anticoagulation in the setting of severe COVID-19 [10].

Sometimes, a D-dimer level can be helpful to demonstrate hypercoagulability in the setting of COVID-19. Several case reports have documented thrombosis in the setting of COVID-19 and a positive D-dimer [11,12]. In our patient’s case, a D-dimer was not obtained at the first ED visit. A CT angiography (CTA) of the chest, however, was done and did not demonstrate a PE. Hypercoagulability due to COVID-19 is postulated to occur secondary to endothelial cell damage and elevation in proinflammatory cytokines [11,13,14]. Thromboses due to COVID-19 hypercoagulability are treated with anticoagulation as they would be due to any other cause.

The patient in this case report had a history of DVT/PE with an IVC filter and bilateral vascular stenting; whether anticoagulation would have prevented this severe complication of COVID-19 infection is uncertain. Amid the COVID-19 pandemic, clinicians must be aware of thrombotic complications of the disease and should consider anticoagulation in high-risk patients.

## Conclusions

Hypercoagulability is an increasingly common complication among COVID-19 patients. There is limited data on the utility of prophylactic and therapeutic anticoagulation in these patients, but it is important to consider its use in high-risk patients. Phlegmasia cerulea dolens is a rare complication, and clinicians should keep this on their differential when evaluating a patient suspected of having COVID-19.
